# Face masks impair facial emotion recognition and induce specific emotion confusions

**DOI:** 10.1186/s41235-022-00430-5

**Published:** 2022-09-05

**Authors:** Mike Rinck, Maximilian A. Primbs, Iris A. M. Verpaalen, Gijsbert Bijlstra

**Affiliations:** Behavioural Science Institute, Radboud University Nijmegen, PO Box 9104, 6500 HE Nijmegen, The Netherlands

**Keywords:** Face masks, Facial emotion recognition, Radboud Faces Database

## Abstract

Face masks are now worn frequently to reduce the spreading of the SARS-CoV-2 virus. Their health benefits are undisputable, but covering the lower half of one's face also makes it harder for others to recognize facial expressions of emotions. Three experiments were conducted to determine how strongly the recognition of different facial expressions is impaired by masks, and which emotions are confused with each other. In each experiment, participants had to recognize facial expressions of happiness, sadness, anger, surprise, fear, and disgust, as well as a neutral expression, displayed by male and female actors of the Radboud Faces Database. On half of the 168 trials, the lower part of the face was covered by a face mask. In all experiments, facial emotion recognition (FER) was about 20% worse for masked faces than for unmasked ones (68% correct vs. 88%). The impairment was largest for disgust, followed by fear, surprise, sadness, and happiness. It was not significant for anger and the neutral expression. As predicted, participants frequently confused emotions that share activation of the visible muscles in the upper half of the face. In addition, they displayed response biases in these confusions: They frequently misinterpreted disgust as anger, fear as surprise, and sadness as neutral, whereas the opposite confusions were less frequent. We conclude that face masks do indeed cause a marked impairment of FER and that a person perceived as angry, surprised, or neutral may actually be disgusted, fearful, or sad, respectively. This may lead to misunderstandings, confusions, and inadequate reactions by the perceivers.

## Highlights

1. We studied how facial masks impair facial emotion recognition.
2. We used validated actors and facial expressions from the Radboud Faces Database.
3. Masks impaired recognition of disgust, fear, surprise, sadness, and happiness.
4. With masks, disgust was often misinterpreted as anger, and fear as surprise.
5. These results were replicated twice in large samples.

## Significance statement

During the COVID-19 pandemic, people got used to wearing protective face mask and to seeing others wear them. The health benefits of face masks are indisputable, but covering the lower half of one's face also makes it harder for others to recognize emotions. This overall effect is quite obvious, but will people recognize all emotions less correctly? Which mistakes will they make when they have to recognize emotions on masked faces? Will they make all kinds of mistakes? Or will they confuse only certain emotions with each other? In order to answer these questions, we conducted three online experiments. In each experiment, participants saw faces that expressed happiness, sadness, anger, surprise, fear, or disgust, or a neutral expression. On half of the faces, the lower part of the face was covered by a face mask. In all experiments, emotion recognition from masked faces was about 20% worse than from unmasked faces. Many mistakes occurred for disgust, followed by fear, surprise, sadness, and happiness. Recognition of anger and the neutral expression was hardly affected. As expected, participants frequently confused emotions that looked similar in the upper half of the face. However, these confusions did not go both ways: Masked disgust was misinterpreted as anger, and fear as surprise, and sadness as neutral, whereas the opposite misinterpretations were less frequent. Therefore, the next time you see a masked person that seems to be angry, surprised, or neutral, be aware that the person may actually be disgusted, fearful, or sad, respectively.

Masks save lives. During the current COVID-19 pandemic, wearing a protective face mask significantly reduces the risk of contracting and spreading the SARS-CoV-2 virus, thereby reducing the risk of infecting oneself or others with a potentially life-threatening disease (Chu et al., 2020). Therefore, many governments around the world have enforced regulations requiring their citizens to wear surgical face masks or similar devices in public. The positive health effects of these masks notwithstanding, it remains to be examined how they affect other aspects of human life, including communication. For instance, individuals with hearing problems may be strongly affected because face masks prevent lip reading. Here, we focus on an important social aspect of communication: facial emotion recognition (FER). In short, can we still recognize the emotions displayed by others if the lower half of their face is covered by a mask? And if emotion recognition is indeed compromised by face masks, which emotions are affected the most, and which emotions are confused with each other most often? The more people are wearing face masks, the more important it is to find answers to these questions. Until recently, in large parts of the world, masks were mainly worn by hospital staff. Nowadays, with masks being present in many contexts, the problem is multiplied: Is the train passenger looking fearful or surprised, is the store customer angry or disgusted, is the neighbor sad or looking neutrally? In all of these cases, a misunderstanding of the expressed emotion may lead to problems in communication.

There is a quickly growing body of research that aims to answer these questions. Studies in which (parts of) the lower half of the face was occluded demonstrated that emotion recognition was impaired, but usually did not establish and confirm which emotions were confused (e.g., Aguado et al., 2009; Schurgin et al., 2014; Yan et al., 2016). More specifically, recent work addressed the overall effect of face masks on FER in adults, finding that face masks do indeed increase the number of recognition errors. This was recently summarized in a review by Pavlova and Sokolov (2022). They concluded that face masks significantly reduce FER accuracy, but that it remains above chance level. Recent work not included in this review (Kastendieck et al., 2022; Parada-Fernández et al., 2022; Tsantani et al., 2022) formulated the same conclusion. For example, Carbon (2020) reported an overall reduction of FER accuracy from 90% unmasked to 73% masked (chance level was 16.7%), with large differences between emotions (disgust 50% reduction, happiness 24%, anger 14%, sadness 13%, neutral 0%, fear − 1%).

Moreover, to better understand how communication is affected by face masks, it is important to investigate these recognition errors in more detail, for example, to find out which emotions are confused with each other. Langbehn et al. (2022) found that FER was reduced for masked faces in dynamically expressed emotions, but this study only included happy, surprised, disgusted, and angry faces. Marini et al. (2021) and Calbi et al. (2021) also described confusions in a limited set of expressions (neutral, happy, sad, and fearful faces, and neutral, happy, and angry faces, respectively). The most extensive set of expressions in which confusions of masked faces were examined, existed of neutral, happy, sad, fearful, disgusted, and angry faces (Blazhenkova et al., 2022, Carbon, 2020; Carbon et al., 2022; Grahlow et al., 2022). These studies converged on the finding that disgust was misinterpreted as anger. However, they show a scattered pattern of confusions for other emotions, e.g., anger being misinterpreted as neutral (Carbon, 2020) or disgust (Grahlow et al., 2022); sadness being misinterpreted as neutral (Carbon, 2020), disgust (Blazhenkova et al., 2022; Grahlow et al., 2022) or fear (Blazhenkova et al., 2022).

This accumulation of recent work converges on the conclusion that face masks impede emotion recognition, while it remains equivocal which emotions are affected the most, and which emotions are confused with each other most often. When we focus on the work describing confusions in masked faces, none of these studies included surprise as an emotion, even though it is one of the basic emotions (Ekman & Friesen, 1971). Indeed, Blazhenkova et al. (2022) included “surprise” as an answer option and found that happiness and fear were misinterpreted as surprise above chance level. This illustrates that limited answer categories may have led to artificial agreements, as none of the studies included "other" as a response option (Frank & Stennett, 2001). Moreover, direct replications are missing, which is important given that the analyses of FER patterns were usually exploratory. Only Carbon et al. (2022) reported confirmatory tests of the hypothesis that recognition of emotions for which the mouth area was indicative would deteriorate the most. Thus, even though a fairly large and growing body of evidence is available already, more detailed and more robust research is needed to advance our insight into FER impairment patterns in masked faces.

Therefore, we conducted a series of three large-scale experiments, comparing the participants' FER performance for masked faces to that for unmasked faces. Most importantly, to complement earlier research, the second and third experiment were designed to test whether the observed results can be replicated with different stimuli and participant groups, all experiments included "other" as a response option, and all experiments focused on the observed confusions between emotion pairs. As emotional face stimuli, we used images from the Radboud Faces Database (RaFD; Langner et al., 2010). These stimuli are well-validated (Bijsterbosch et al., 2021; Langner et al., 2010; Mishra et al., 2018; Verpaalen et al., 2019). Moreover, the actors were instructed to express the emotions using the "Facial Action Coding System" (FACS; Ekman et al., 2002), which is based on the contraction and relaxation of emotion-specific facial muscles. Therefore, the visual similarity of different emotions can be defined by comparing their specific patterns of activated muscles, so-called action units. For instance, the action units surrounding the eyes involved in the expressions of fear and surprise overlap, and indeed these two emotions are often confused, as are anger and disgust (Langner et al., 2010; Susskind et al., 2007). The idea that emotional expressions are easily confused if they involve similar muscle movements is not new; it has also been mentioned as part of the “perceptual-attentional limitation hypothesis” (Chamberland et al., 2017; Roy-Charland et al., 2014). Most important here, the facial muscles can be divided into those covered by face masks versus those that remain visible. Thus, from a theoretical point of view, we can predict that face masks will increase confusions particularly between those emotions that differ in the activation of covered muscles, but share activation of the muscles in the upper half of the face (see also Smith et al., 2005; Wegrzyn et al., 2017).

## Experiment 1

Based on these assumptions, we pre-registered the obvious prediction that masks will impair FER performance, plus the more specific and theory-based prediction that face masks will increase confusions of anger and disgust, as well as confusions of fear and surprise. To test these predictions, we presented images of male and female actors from the RaFD to the participants. Each image displayed one of the following FACS-generated emotional expressions: happiness, sadness, anger, surprise, fear, or disgust, or a neutral expression. Moreover, each image was presented twice: once with a face mask, once without.

## Methods

### Participants

As pre-registered, we recruited 100 participants who fulfilled our inclusion criteria (at least 18 years old, fluent in English) and completed the online study via the research platform *Prolific*. Of those, 9 fulfilled our pre-registered exclusion criteria (not rating all facial stimuli; not participating seriously; overall raw hit rate significantly lower than the median raw hit rate; not finishing the study within one hour; taking significantly longer or shorter than the median completion time; self-reported demographic data not matching the inclusion criteria), leaving the data of 91 participants to be analyzed. Their mean age was 33.23 years (*SD* = 10.42), all but one had English as their native language (the remaining one had Greek), 23 were male, 67 female, and 1 non-binary. Their current country of residence was UK (83), USA (4), Ireland (2), Denmark (1), or Germany (1). The majority of them (57) reported that in their region of residence, wearing masks was only obligatory in certain situations, 29 reported that wearing masks was not required, and 5 reported that wearing masks was obligatory outside. The statistical power achieved by the current sample size (N = 91) was computed using the program G*Power 3.1 (Faul et al., 2009). For the main effect of mask condition on recognition accuracy described below, this yielded excellent power of 1 − *ß* > 0.99 to detect a medium-sized effect (*f* = 0.25; *p* = 0.05, *r* = 0.50), and insufficient power of 1 − *ß* = 0.47 to detect a small effect (*f* = 0.10; *p* = 0.05, *r* = 0.50). Participants received information about the study and gave informed consent before participating. They received £ 2.25 as compensation for their participation. We preregistered the sample size, hypotheses, and statistical analyses of Experiment 1 at the Open Science Framework (https://osf.io/3d647/), where the anonymized data and the analysis scripts are also available. All three experiments of the current study were conducted in accordance with the code of ethics of the World Medical Association (Declaration of Helsinki) for experiments involving humans. Furthermore, the study was independently reviewed and approved by the local ethics committee (#ECSW-2020-074).

### General procedure

After signing up for the study via *Prolific*, participants were directed to the FER task programmed in Qualtrics. Participants first received information about the task and gave informed consent, then completed the two blocks of 84 trials each of the task, described in detail below. Afterward, they completed a seriousness check by choosing between "I have taken part seriously" or "I have just clicked through, please throw my data away" (Aust et al., 2013). No one chose the second option. They were then asked to give information about their gender, age, native language, and country of residence. This was followed by the mask experience questions described below. The whole procedure took approximately 15–25 min, depending on the individual speed of the participants.

### Materials

**Fig. 1:**
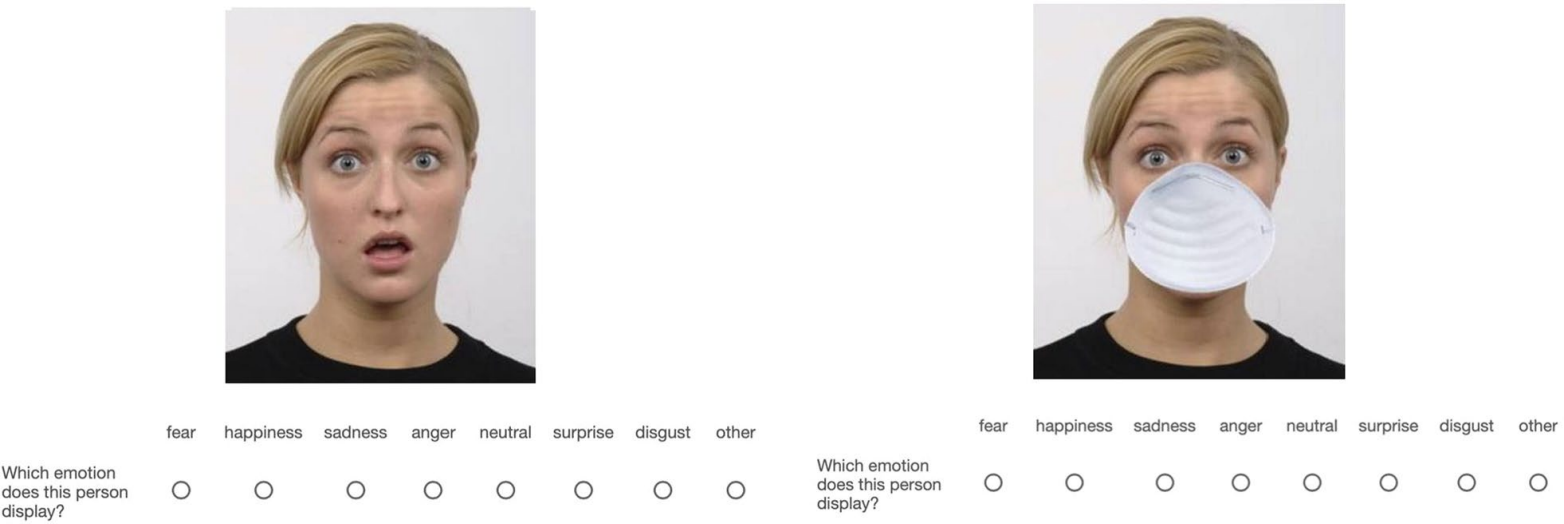
Sample trials without versus with mask (here: surprise)

*Pictures* We selected portraits of 12 different actors (6 males, 6 females) from the Radboud Faces Database (RaFD; Langner et al., 2010). Frontal views of the following actors were used: 01, 02, 03, 04, 07, 08, 12, 22, 46, 47, 49, 71. These actors were selected because none of their emotional expressions had an agreement rate lower than 10% from the mean, within the original validation study (Langner et al., 2010). Each actor showed each of the 7 facial emotional expressions (happiness, sadness, anger, surprise, fear, disgust, neutral), yielding 84 different images. From each original image, a masked version was created by superimposing the picture of a face mask over the lower part of the face, covering the mouth-nose region (see Fig. 1). This yielded a total of 168 different images to be displayed. All pictures were 280 × 350 pixels large and presented in color.

*Mask experience questions* Participants answered 3 questions related to their everyday experience with masked faces. (1) "The last time you went out to buy groceries or something else, how many people did you see wearing masks?" They responded by choosing a value between 0 (*none*) and 100 (*everyone*). (2) "What is the current policy regarding face masks in the region you reside in?" Participants could choose between the 3 answer options "Wearing a mask is obligatory outside", "Wearing a mask is only obligatory in certain situations, like when in school or on the train", and "Wearing a mask is not required". (3) "Did people around you wear masks before the COVID-19/Corona virus in the past years?" They responded by choosing a value between 0 (*no one in your daily environment*) and 100 *(everyone in your daily environment*). Finally, they were asked to type in any other comments they might have about the study.

### Facial emotion recognition task

The task was programmed in Qualtrics, consisting of a total of 168 trials, which resulted from the full combination of four factors: 7 emotional expressions (fear, happiness, sadness, anger, neutral, surprise, disgust) by 2 mask conditions (masked, unmasked) by 2 genders (male, female) by 6 actors. The trials were split into two fixed blocks of 84 trials each (7 expressions by 2 genders by 6 actors in each block), such that each actor was presented with a specific expression once in each block. In each block, half of the images were presented in their original version without mask, and the other half with mask. This way, each block included three images for each emotion x mask x gender combination (7 × 2x2 × 3 = 84). The order of the two blocks was counterbalanced across participants. Within each block, the order of the 84 images was random. Figure 1 illustrates the nature of each trial: Participants were shown one image and asked to recognize the displayed emotion from the face, either without mask (Fig. 1, left) or with mask (Fig. 1, right). They responded by choosing one of the 8 answer options, which were presented in the order fear, happiness, sadness, anger, neutral, surprise, disgust, other (see Fig. 1) for half of the participants, and in the order disgust, surprise, neutral, anger, sadness, happiness, fear, other, for the other half. Participants did not receive feedback after their response, but a response had to be made in order to continue.

### Data preparation and analyses

*Hit rates and arcsine-transformed rates* Hit rates (mean percentages of correct responses) were first computed separately for each displayed emotion and mask condition. These rates indicate the degree to which recognized emotions were identical to the emotions as expressed by the actors. Second, to correct for the skewed variances of the decimal fractions obtained from the counts, the hit rates were arcsine-transformed (Winer, 1971).

*Unusual response patterns* We also checked whether the data contained unusual response patterns, for instance, whether any participant always responded with a specific emotion category. This was not the case; no participant showed an unusual response pattern.

*Analyses* First, to determine the effect of face masks on FER performance, we performed a repeated-measures analysis of variance (ANOVA) on the arcsine-transformed hit rates, with expression (fear, happiness, sadness, anger, neutral, surprise, disgust) and mask condition (unmasked, masked) as within-subjects factors. To further explore the interaction, we performed post hoc paired *t*-tests with corrections for multiple testing (Holm, 1979). Second, we explored the pattern of confusions, separately for masked and unmasked faces. To this end, we first tested for each of the 7 displayed emotions whether the confusions were systematically distributed, that is, if any of the 7 incorrect response options was chosen more often than expected by chance. The confusion chance level was computed as: (100% − %correct)/7. Next, we tested our predictions that masks would increase confusions of anger with disgust and vice versa, as well as confusions of fear with surprise and vice versa. The analyses relied on the statistical analysis software JASP (version 0.13.1; JASP Team, 2020) and the following software packages implemented in R (R Core Team, 2020): tidyverse (version 1.3; Wickham et al., 2019), plyr (version 1.8.6; Wickham, 2011); gmodels (version 2.18.1; Warnes et al., 2018), MASS (version 7.3.51.6; Venables & Ripley, 2002), dplyr (Wickham et al., 2020) and janitor (version 2.0.1; Firke, 2020).

*Transparency statement* We deviated from the pre-registered analyses in two points: First, we pre-registered that we would compute the arcsine-transformed hit rates based on the unbiased hit rates. Unfortunately, the unbiased hit rates could not be computed as planned, because several hit rates (13 in total) equaled 0. Hence, the computation of the unbiased hit rates would have involved a division by 0. Therefore, we calculated the arcsine-transformed hit rates based on the raw hit rates rather than the unbiased hit rates (the same had to be done in Experiments 2A and 2B). Second, we pre-registered that we would conduct a two-sample chi-square test to compare the number of confusions between the mask and no-mask condition and conduct follow-up *t*-tests. Instead, to avoid alpha inflation, we immediately conducted the follow-up *t*-tests, and only for emotions where the actual count exceeded the count expected by chance for either the masked or unmasked condition (the same was done for Experiments 2A and 2B).

## Results

### Recognition accuracy

Table 1 shows how often each answer alternative was chosen when the 7 different emotional expressions were shown with vs. without a mask. The cells marked in light gray show correct responses, that is, the mean raw hit rates and their standard deviations. The corresponding arcsine-transformed hit rates were analyzed using a 7 × 2 ANOVA with expression (fear, happiness, sadness, anger, neutral, surprise, disgust) and mask condition (unmasked, masked) as within-subjects factors. The ANOVA revealed the predicted detrimental effect of masks on FER: On average, participants recognized the emotions correctly from 87.9% of the unmasked faces, compared to only 69.2% of the masked ones, *F*(1,90) = 788.42, *p* < 0.001, η_p_^2^ = 0.90. Moreover, we found a significant main effect of emotion, *F*(6,540) = 141.998, *p* < 0.001, *η*_p_^2^ = 0.61. Notably, the detrimental effect of masks was not the same for all emotions, however (see Table 1), yielding a significant mask * emotion interaction, *F*(6,540) = 74.77, *p* < 0.001, *η*_p_^2^ = 0.45. The most dramatic reduction in FER was observed for disgust (minus 57%), *t*(90) = 27.35, *p* < 0.001, *dz* = 2.86. The reduction was also significant for fear (minus 27%), *t*(90) = 10.31, *p* < 0.001, *dz* = 1.08, for sadness (minus 22%), *t*(90) = 12.58 *p* < 0.001, *dz* = 1.38, for surprise (minus 9%), *t*(90) = 9.81, *p* < 0.001, *dz* = 0.99, and for happiness (minus 7%), *t*(90) = 7.31, *p* < 0.001, *dz* = 0.79. In contrast, it was not significant for anger (minus 2%), *t*(90) = 1.67, *p* > 0.99, *dz* = 0.16, and for the neutral expression (minus 2%), *t*(90) = 1.53, *p* > 0.99, *dz* = 0.18.

**Table 1:** Choice percentages (with SDs) per mask condition, displayed and chosen emotion category, in Experiment 1

Mask and displayed emotion	Chosen emotion								
	Fear	Happiness	Sadness	Anger	Neutral	Surprise	Disgust	Other	Chance
No mask									
Fear	*65.29* *(25.16)*	0.00 (0.00)	0.82 (2.79)	0.55 (2.08)	0.37 (2.12)	24.63*** (19.76)	5.22 (9.91)	3.11 (8.49)	4.96
Happiness	0.00 (0.00)	*99.45* *(2.08)*	0.09 (0.87)	0.00 (0.00)	0.00 (0.00)	0.09 (0.87)	0.09 (0.87)	0.28 (1.50)	0.08
Sadness	0.37 (1.72)	0.18 (1.23)	*92.58* *(9.33)*	1.28 (4.11)	2.56*** (4.06)	0.00 (0.00)	1.37 (3.57)	1.65 (5.30)	1.06
Anger	1.19 (3.42)	0.00 (0.00)	5.77** (8.85)	*76.92* *(19.25)*	0.73 (2.95)	0.73 (3.20)	6.78** (9.93)	7.88*** (12.69)	3.30
Neutral	0.00 (0.00)	1.47* (3.19)	2.47** (5.89)	0.64 (3.10)	*94.51* *(9.06)*	0.09 (0.87)	0.09 (0.87)	0.73 (2.95)	0.78
Surprise	4.03*** (7.59)	0.00 (0.00)	0.09 (0.87)	0.09 (0.87)	0.37 (1.72)	*93.77* *(8.48)*	0.64 (2.56)	1.01 (3.00)	0.89
Disgust	0.18 (1.23)	0.00 (0.00)	0.00 (0.00)	5.50*** (9.15)	0.09 (0.87)	0.55 (2.42)	*92.58* *(11.28)*	1.10 (3.77)	1.06
With mask									
Fear	*38.37* *(26.03)*	0.09 (0.87)	2.47 (4.40)	0.37 (1.72)	0.28 (1.50)	56.69*** (25.10)	1.10 (3.10)	0.64 (2.56)	8.80
Happiness	0.00 (0.00)	*92.31* *(9.72)*	0.09 (0.87)	0.09 (0.87)	6.14*** (8.13)	0.55 (2.08)	0.09 (0.87)	0.73 (3.20)	1.10
Sadness	4.76 (7.36)	0.00 (0.00)	*70.79* *(21.46)*	4.76 (5.85)	10.35*** (13.40)	1.28 (3.50)	4.21 (6.96)	3.85 (9.49)	4.17
Anger	2.01 (4.54)	0.00 (0.00)	5.13* (7.33)	*75.09* *(17.89)*	1.10 (3.33)	0.55 (2.42)	13.19*** (12.18)	2.93 (9.65)	3.56
Neutral	0.09 (0.87)	1.28 (3.02)	3.39*** (6.08)	1.47 (6.16)	*92.86* *(10.14)*	0.18 (1.23)	0.09 (0.87)	0.64 (2.23)	1.02
Surprise	8.06*** (9.74)	0.46 (1.91)	2.20 (4.27)	0.09 (0.87)	6.59** (10.43)	*79.49* *(15.53)*	0.64 (2.23)	2.47 (7.81)	2.93
Disgust	0.09 (0.87)	2.29 (4.98)	1.47 (3.85)	49.73*** (21.24)	8.24 (6.27)	0.55 (2.42)	*35.35* *(19.97)*	2.29 (6.58)	9.24

The cells show raw choice percentages instead of the analyzed arcsine transformed percentages. Hit rates are marked in italic. Asterisks indicate confusion percentages above the chance level shown in the right column (* *p* < .05, ** *p* < .01, *** *p* < .001). Chance levels refer to the distribution of confusions only, excluding the hits. Percentages are rounded to two digits and may not add up to exactly 100%

### Confusions

Table 1 also shows the incorrect responses, that is, the confusions. Separately for masked vs. unmasked faces and for each emotional expression, we first analyzed whether these incorrect responses were distributed equally across the 7 answer alternatives (indicating random guessing), or whether some alternatives were chosen significantly more often than expected by chance (indicating systematic confusions).

Table 1 shows that systematic confusions almost always occurred, except for unmasked happiness which did not leave room for confusions because it was recognized almost 100% correct. Most importantly for our predictions, when fear was misinterpreted, most often it was taken for surprise. This happened significantly more often than expected by chance both for unmasked faces (approx. 25%) and masked ones (approx. 57%). In fact, with masks, this confusion occurred even more often than the correct interpretation (approx. 38%), *t*(90) = 3.30, *p* = 0.001, *dz* = 0.35. As predicted, the confusion of fear and surprise also occurred the other way around, albeit less often. Both with and without masks, fear was the dominant incorrect response to surprised faces (approx. 8% and 4%, respectively). With masks, surprise was also sometimes interpreted as neutral (approx. 7%).

Similar reciprocal confusions in line with our predictions were observed for anger and disgust (see Table 1). When unmasked anger was confused, it was confused most often with disgust or sadness (approx. 7% and 6%, resp.), just like masked anger was (approx. 13% and 5%, resp.). In addition, unmasked anger was misinterpreted as "other" (approx. 8%). The other way around, when unmasked disgust was confused, it was interpreted most often as anger (approx. 5%). When disgust was masked, this confusion occurred extremely often (approx. 50%), even more often than the correct interpretation (approx. 35%), *t*(90) = 3.50, *p* < 0.001, *dz* = 0.37.

When exploring the confusion patterns further, we found that sadness and the neutral expression also showed evidence of reciprocal above-chance level confusions. Both unmasked and masked sadness, when confused, were misinterpreted as neutral (approx. 3% and 10%, resp., see Table 1). Conversely, when the unmasked or masked neutral expression was misinterpreted, participants confused it with sadness most often (approx. 2% and 3%, resp., see Table 2).

**Table 2:** Choice percentages per mask condition, displayed and chosen emotion category, in Experiment 2A

Mask and displayed emotion	Chosen emotion								
	Fear	Happiness	Sadness	Anger	Neutral	Surprise	Disgust	Other	Chance
No mask									
Fear	*66.95* *(26.55)*	0.19 (1.24)	1.97 (3.98)	0.37 (2.14)	0.47 (3.63)	16.20*** (19.40)	10.49*** (15.31)	3.37 (9.87)	4.72
Happiness	0.00 (0.00)	*99.44* *(2.45)*	0.09 (0.88)	0.00 (0.00)	0.47 (2.30)	0.00 (0.00)	0.00 (0.00)	0.00 (0.00)	0.08
Sadness	1.78 (4.26)	0.00 (0.00)	*91.48* *(11.51)*	1.03 (3.03)	1.50 (5.41)	0.47 (2.30)	1.69 (5.78)	2.06 (4.40)	1.22
Anger	1.03 (2.76)	0.00 (0.00)	3.37 (6.85)	*77.81* *(20.95)*	5.24* (7.47)	1.22 (4.27)	6.74** (11.09)	4.59 (10.59)	3.17
Neutral	0.37 (1.74)	0.75 (2.40)	2.72* (7.19)	2.25* (4.83)	*92.79* *(9.75)*	0.19 (1.77)	0.28 (1.97)	0.66 (3.13)	1.03
Surprise	3.84** (7.95)	0.09 (0.88)	0.19 (1.24)	0.09 (0.88)	0.19 (1.77)	*92.32* *(10.38)*	2.34* (4.86)	0.94 (3.19)	1.10
Disgust	0.28 (1.97)	0.00 (0.00)	0.00 (0.00)	9.93*** (13.41)	0.19 (1.77)	0.75 (3.24)	*86.89* *(15.84)*	1.97 (6.88)	1.87
With mask									
Fear	*32.68* *(21.59)*	0.37 (1.74)	2.06 (4.40)	1.87 (4.83)	1.69 (4.90)	57.77*** (20.79)	2.34 (4.35)	1.22 (3.67)	9.62
Happiness	0.09 (0.88)	*89.42* *(12.17)*	0.47 (1.93)	0.56 (2.10)	6.84*** (9.78)	0.19 (1.24)	1.22 (2.96)	1.22 (3.88)	1.51
Sadness	11.14*** (12.31)	0.47 (1.93)	*59.18* *(23.54)*	1.97 (4.53)	14.98*** (18.85)	2.90 (6.66)	3.93 (6.42)	5.43 (10.74)	5.83
Anger	2.06 (5.66)	0.28 (1.97)	4.40 (7.86)	*72.38* *(20.44)*	5.52 (8.70)	1.22 (4.27)	11.61*** (13.57)	2.53 (5.24)	3.95
Neutral	0.84 (2.82)	0.75 (2.98)	2.06 (6.32)	2.90* (6.42)	*90.64* *(12.80)*	1.12 (3.37)	0.56 (2.45)	1.12 (4.90)	1.34
Surprise	9.55*** (11.96)	1.12 (3.13)	1.87 (4.83)	0.47 (1.93)	5.15* (9.44)	*78.56* *(17.13)*	1.40 (3.82)	1.87 (4.83)	3.06
Disgust	0.84 (3.09)	9.08 (8.85)	2.25 (4.83)	53.37*** (22.25)	3.18 (6.59)	1.12 (4.39)	*27.43* *(19.83)*	2.72 (6.00)	10.37

The cells show raw choice percentages instead of the analyzed arcsine transformed percentages. Hit rates are marked in italic. Asterisks indicate confusion percentages above the chance level shown in the right column (* *p* < .05, ** *p* < .01, *** *p* < .001). Chance levels refer to the distribution of confusions only, excluding the hits. Percentages are rounded to two digits and may not add up to exactly 100%

Additional analyses were conducted to test the predicted increases in anger-disgust confusions and fear-surprise confusions caused by masks. The results showed that these did indeed occur as predicted, but to different degrees. Misinterpretations of disgust as anger became much more frequent with masks (approx. 5% vs. 50%), *t*(90) = 20.64, *p* < 0.001, *dz* = 1.93, whereas the reversed misinterpretation of anger as disgust was less frequent and did not increase as dramatically (approx. from 7 to 13%), *t*(90) = 4.92, *p* < 0.001, *dz* = 0.52. A similar asymmetry was observed for fear and surprise: Without masks, misinterpretations of fear as surprise were far less frequent than with masks (approx. 25% vs. 57%), *t*(90) = 14.07, *p* < 0.001, *dz* = 1.39, whereas the reversed misinterpretation of surprise as fear was less frequent and did not increase as dramatically with masks (approx. from 4 to 8%), *t*(90) = 3.61, *p* < 0.001, *dz* = 0.37. Interestingly, masked faces revealed a strongly asymmetric pattern of confusions, favoring anger and surprise: Masked disgust was taken for anger more often than masked anger was taken for disgust, *t*(90) = 12.06, *p* < 0.001 *dz* = 1.26, and masked fear was taken for surprise more often than masked surprise was taken for fear, *t*(90) = 14.04, *p* < 0.001, *dz* = 1.47.

### Exploratory analyses

*Model gender* Given that we presented only 6 actors per gender, we had no specific hypotheses regarding interactions or a main effect of model gender. We did not observe a significant main effect of gender, as female (79.7%) and male (77.4%) faces were recognized similarly well, *F*(1,90) = 1.98, *p* = 0.162, *η*_p_^2^ = 0.022. The gender * mask interaction was non-significant, *F*(1,90) = 1.78, *p* = 0.185, *η*_p_^2^ = 0.01, suggesting that the detrimental effect of masks did not differ between male and female faces. In contrast, the emotion * gender interaction, *F*(6, 540) = 10.34, *p* < 0.001, *η*_p_^2^ = 0.10, and the mask * emotion * gender three-way interaction, *F*(6,540) = 7.507, *p* < 0.001, *η*_p_^2^ = 0.08, were significant. Inspection of the means revealed that the latter interaction occurred because for male faces, masks did not reduce recognition of angry and neutral expressions, whereas for female faces, all emotions were affected.

*Experience with face masks* We had no previous hypotheses about this factor. The participants' ratings of how many people they saw wearing masks last time they went out to buy something (between 0 [*none*] and 100 [*everyone*] correlated significantly with their recognition performance. Unexpectedly, the more masks they reported having seen, the worse their performance was for both unmasked faces, *r*(89) = − 0.277, *p* = 0.008, and masked faces, *r*(89) = − 0.212, *p* = 0.043. However, the detrimental effect of masks did not correlate with mask experience, *r*(89) = − 0.043, *p* = 0.683. The answers to the question "Did people around you wear masks before the COVID-19/Corona virus in the past years?" did not correlate with any aspect of recognition performance, all *r*(81) < − 0.10, *p* > 0.38. The mean value of the answers was extremely low, averaging 2.73 on a scale ranging from 0 to 100.

Before speculating on potential explanations of the exploratory findings, we aimed to replicate them in the following experiments, because they may be specific to the small sample of stimuli used here.

## Discussion

The main goal of Experiment 1 was to find out if and how strongly the recognition of seven different facial emotional expressions is impaired by face masks. Moreover, we tested our prediction that if face masks increase confusions of emotions, then it would be particularly those emotions that have similar activation patterns of the face muscles in the uncovered upper half of the face, namely anger and disgust as well as fear and surprise.

The results of the experiment were mostly in line with our pre-registered predictions. First, although the current study used only 12 of the original RaFD actors, the recognition accuracies for unmasked faces observed here compare fairly well to the accuracies reported by Langner et al. (2010) and other validation studies. Happiness was recognized almost perfectly, and high hit rates above 90% were also observed for sadness, surprise, disgust, and the neutral expression. Only fear and anger were recognized less well. Differences between the current recognition accuracies and previously reported ones may be due to the online format we used, to differences in sample demographics, and to the fact that we presented only a small subsample of the previously used actors. Second, as predicted, recognition of the emotional expressions was impaired by face masks: On average, correct recognitions were reduced from about 88% to about 69%. The size of the impairment varied greatly across emotions, with the most dramatic reduction observed for disgust, followed by fear, sadness, surprise, and happiness. In contrast, it was not significantly reduced for anger or the neutral expression.

Third, when emotions were misinterpreted, the pattern of confusions followed our prediction that masks would increase confusions of those emotions that involve similar activations of the muscles in the unmasked upper half of the face. Indeed, we found that masks increased confusions of anger and disgust with each other, as well as fear and surprise with each other. In fact, with masks, some confusions were even more frequent than the correct response: Masked fear was interpreted incorrectly as surprise more often than correctly as fear, and masked disgust was interpreted incorrectly as anger more often than correctly as disgust. In addition, and unrelated to our predictions, we found that the sad expression and the neutral expression were also confused with each other, and more so with masks than without. A potential post hoc explanation may be that they do indeed look somewhat similar on the RaFD faces: Sadness is expressed rather mildly because tears are missing, and the neutral expression looks fairly stern and "empty" because no muscles are activated.

Unexpectedly, the observed confusion patterns were asymmetric: Masks caused fear to be misinterpreted as surprise much more often than surprise was misinterpreted as fear, and disgust was misinterpreted as anger much more often than anger was misinterpreted as disgust. The latter result may also explain why masks did not reduce the overall recognition rate of anger. It seems that participants had a response bias: When seeing a masked angry or disgusted face, they rather responded with "anger" than with "disgust" or any other emotion. We can only speculate about the potential reasons why surprise was a more likely response than fear, and anger more likely than disgust. However, before doing so, we decided to test whether the current results would replicate with new samples of materials and participants. To that end, we conducted Experiments 2A and 2B reported below.

## Experiments 2A and 2B

Since Experiments 2A and 2B were very similar, and both were designed as replications of Experiment 1, they are reported together, in order to avoid repetitive descriptions. To test the replicability of the results across materials and participants, we not only recruited new samples, but also replaced the facial stimuli used in the first experiment by equivalent ones from the RaFD. In Experiment 2A, all other aspects of the experiment remained unchanged, including the procedure, the recruitment via Prolific, and the analyses. The experiment was also pre-registered at the Open Science Framework (https://osf.io/86nm7/). Experiment 2B used the same new stimulus set as Experiment 2A. In contrast to Experiments 1 and 2A, however, we did not recruit an international sample of paid participants via *Prolific*. Instead, we recruited students of Radboud University who participated for course credit, thereby yielding a sample that was more homogeneous and more similar to samples of pre-pandemic studies. Most importantly, the sample was also considerably larger than the samples of the first two experiments. This allowed us to return to the question of gender effects, to determine with more statistical power whether the detrimental effect of face masks on FER would be larger for male or female actors, and additionally, for male or female observers.

## Experiment 2A: Methods

### Participants

Of the 101 participants recruited via *Prolific*, 12 fulfilled our pre-registered exclusion criteria (see Experiment 1), leaving the data of 89 participants to be analyzed. Their mean age was 38.66 years (*SD* = 12.09), all had English as their native language, and 29 were male and 60 female. Their current country of residence was the UK (79), USA (5), Ireland (2), Japan (1), Mexico (1), and Poland (1). The majority of them (68) reported that in their region of residence, wearing masks was only obligatory in certain situations, 19 reported that wearing masks was obligatory outside, and a single person reported that wearing masks was not required. The statistical power achieved by the current sample size (*N* = 89) resembled that of the first experiment: For the main effect of mask condition on recognition accuracy, it yielded excellent power of 1 − *ß* > 0.99 to detect a medium-sized effect (*f* = 0.25; *p* = 0.05, *r* = 0.50), and insufficient power of 1-*ß* = 0.46 to detect a small effect (*f* = 0.10; *p* = 0.05, *r* = 0.50).

### Materials

The actors of Experiment 1 were replaced by other actors from the RaFD, namely the female actors 14, 27, 31, 32, 57, 58, and the male actors 20, 23, 24, 25, 28, 33. The pictures of these actors were processed in the same way as the ones used in Experiment 1.

## Experiment 2A: Results

### Recognition accuracy

Table 2 shows how often each answer alternative was chosen in this experiment when the 7 different emotional expressions were shown with vs. without a mask. The cells marked in light gray show correct responses, that is, the mean raw hit rates and their standard deviations. The corresponding arcsin-transformed hit rates were again analyzed using a 7 × 2 ANOVA with expression (fear, happiness, sadness, anger, neutral, surprise, disgust) and mask condition (unmasked, masked) as within-subjects factors. The hit rates replicated the detrimental effect of masks on FER: On average, participants recognized the emotions correctly from 86.8% of the unmasked faces, compared to only 64.3% of the masked ones, *F*(1,88) = 785.61 *p* < 0.001, *η*_p_^2^ = 0.90. In addition, we again observed a significant main effect of emotion, *F*(5, 528) = 127.58, *p* < 0.001, *η*_p_^2^ = 0.59. As in Experiment 1, a significant mask-by-emotion interaction, *F*(6,528) = 66.57, *p* < 0.001, *η*_p_^2^ = 0.43, indicated that the effect of masks was larger for some emotions than for others (see Table 2). The largest reduction in FER was observed for disgust (minus 59%), *t*(88) = 22.79, *p* < 0.001, *dz* = 2.42. The reduction was also significant for fear (minus 34%), *t*(88) = 12.68, *p* < 0.001, *dz* = 1.34, for sadness (minus 32%), *t*(88) = 18.77, *p* < 0.001, *dz* = 1.99, for surprise (minus 14%), *t*(88) = 7.87, *p* < 0.001 *dz* = 0.83, for happiness (minus 10%), *t*(88) = 9.72, *p* < 0.001, *dz* = 1.03, and for anger (minus 5%), *t*(88) = 3.15, *p* = 0.002 *dz* = 0.33. In contrast, it was negligible for the neutral expression (minus 2%), *t*(88) = 1.00, *p* = 0.32 *dz* = 0.11. The rank order of the reductions is an exact replication of Experiment 1, and the absolute values are similar, but slightly larger here. Moreover, the small FER reduction for angry faces was significant, whereas it had not been significant in Experiment 1.

### Confusions

Table 2 also shows the incorrect responses, that is, the confusions. They were strikingly similar to those found in Experiment 1 (compare Table 1). Most importantly, we replicated the main findings of Experiment 1: First, the confusions were not randomly distributed for any of the masked emotions (see the significance values in the lower half of Table 2). Second, masked fear was again frequently misinterpreted as surprise (approx. 58%). As in Experiment 1, this confusion occurred even more often than the correct interpretation (approx. 33%), *t*(88) = 5.67, *p* < 0.001 *dz* = 0.60. Conversely, the most frequent misinterpretation of masked surprise was fear again (approx. 10%). However, this did not occur nearly as often as the opposite confusion, replicating the asymmetric pattern found in Experiment 1, *t*(88) = 14.68, *p* < 0.001 *dz* = 1.56. Third, the dominant interpretation of masked disgust was the misinterpretation as anger (approx. 53%), which again occurred even more often than the correct interpretation (approx. 27%), *t*(88) = 6.13, *p* < 0.001 *dz* = 0.65. Conversely, the most frequent misinterpretation of masked anger was disgust (approx. 12%). However, as in Experiment 1, masked disgust was taken for anger more often than masked anger was taken for disgust, *t*(88) = 11.71, *p* < 0.001 *dz* = 1.24.

The unexpected reciprocal confusion pattern of sadness and the neutral expression was not replicated: Although "neutral" was the most frequent misinterpretation of masked sadness (approx. 15%), "fear" was also a frequent misinterpretation (approx. 11%), and sadness was not an above-chance misinterpretation of the masked neutral expression (approx. 2%), whereas anger was (approx. 3%).

### Exploratory analyses

*Model gender* As in Experiment 1, we did not observe a significant main effect of gender: Male and female faces were recognized similarly well, *F*(1,88) = 3.42, *p* = 0.068, η_p_^2^ = 0.037. The gender * mask interaction was also non-significant, *F*(1,88) = 0.60, *p* = 0.439, *η*_p_^2^ = 0.007, suggesting again that the detrimental effect of masks did not differ between male and female faces. The emotion * gender interaction was significant again, *F*(6, 528) = 12.46, *p* < 0.001, *η*_p_^2^ = 0.124, and so was the mask * emotion * gender three-way interaction, *F*(6,528) = 4.887, *p* < 0.001, *η*_p_^2^ = 0.053. Inspection of the means showed that the three-way interaction was due to the fact that for female faces, again all emotions were affected, whereas for male faces, masks did not reduce the recognition of neutral expressions.

*Experience with face masks.* Unlike Experiment 1, we did not find a significant correlation of the number of masks participants reported having seen with their FER performance for masked faces or unmasked faces, or with the size of the detrimental effect of masks, all *r*(87) < 0.15, *p* > 0.18.

## Experiment 2B: Methods

### Participants

We recruited 287 participants via *Sona,* the online experiment participation system of Radboud University. Of those, 118 were automatically excluded because they indicated that they had not taken part seriously. Unfortunately, this high number is not unusual because students get credit for the time they spend on a study, independent of the quality of their work. Of the remaining 169 participants, 16 fulfilled our exclusion criteria (see Experiment 1), leaving the data of 153 participants to be analyzed (36 males, 117 females). The majority of them were first-year psychology students who participated for course credit. Their mean age was 21.61 years (SD = 6.37). Reflecting the composition of the student population, most of them (113) had Dutch as their native language, followed by German (31), English (2), Bulgarian (2), and Albanian, Croatian, Polish, Portuguese, and Spanish (1 each). Most of them currently lived in the Netherlands (125), while 25 lived in Germany, and one each lived in Belgium, Croatia, and Switzerland. The majority of them (135) reported that in their region of residence, wearing masks was only obligatory in certain situations, 11 reported that wearing masks was obligatory outside, and 7 reported that wearing masks was not required. The statistical power achieved with the current sample size (N = 153) was considerably better than for the previous experiments: For the main effect of mask condition on recognition accuracy and for the interaction of model gender with the mask effect, it yielded excellent power of 1 − *ß* > 0.99 to detect a medium-sized effect (*f* = 0.25; *p* = 0.05, *r* = 0.50), and sufficient power of 1 − *ß* = 0.69 to detect a small effect (*f* = 0.10; *p* = 0.05, *r* = 0.50).

## Experiment 2B: Results

### Recognition accuracy

Table 3 shows how often each answer alternative was chosen in this experiment when the 7 different emotional expressions were shown with vs. without a mask. Again, the cells marked in light gray show correct responses, that is, the mean raw hit rates and their standard deviations. The corresponding arcsine-transformed hit rates were analyzed using a 7 × 2 × 2 × 2 ANOVA with expression (fear, happiness, sadness, anger, neutral, surprise, disgust), mask condition (unmasked, masked), and actor gender (female, male) as within-subjects factors. Participant gender (female, male) was added as a between-subjects factor, given the larger sample size. The observed hit rates replicated the detrimental effect of masks on FER: On average, participants recognized the emotions correctly from 90.7% of the unmasked faces, compared to only 69.5% of the masked ones, *F*(1,152) = 1022.12, *p* < 0.001, *η*_p_^2^ = 0.87. This detrimental mask effect did not depend on actor gender (females: minus 21%, males: minus 21%), *F*(1,151) = 0.18, *p* = 0.68, *η*_p_^2^ = 0.001, or participant gender (females: minus 21%, males: minus 22%), *F*(1,151) = 0.04, *p* = 0.84, *η*_p_^2^ < 0.001. The previously observed emotion * mask * actor gender interaction was significant again, *F*(6,906) = 7.96, *p* < 0.001, η_p_^2^ = 0.05, whereas the corresponding emotion * mask * participant gender interaction was not significant, *F*(6,906) = 1.373, *p* = 0.223, *η*_p_^2^ = 0.009.

**Table 3:** Choice percentages per mask condition, displayed and chosen emotion category, in Experiment 2B

Mask and displayed emotion	Chosen emotion								
	Fear	Happiness	Sadness	Anger	Neutral	Surprise	Disgust	Other	Chance
No mask									
Fear	*75.05* * (21.15)*	0.05 (0.67)	1.69 (4.10)	0.44 (2.09)	0.22 (1.64)	9.42*** (13.03)	10.46*** (11.77)	2.67 (7.00)	3.56
Happiness	0.05 (0.67)	*98.97* *(3.22)*	0.44 (1.86)	0.00 (0.00)	0.22 (1.64)	0.22 (1.33)	0.05 (0.67)	0.05 (0.67)	0.15
Sadness	1.20 (3.23)	0.22 (1.33)	*93.95* *(8.62)*	1.20 (4.10)	0.60 (2.88)	0.38 (1.99)	1.42 (3.68)	1.03 (3.22)	0.86
Anger	0.33 (1.62)	0.00 (0.00)	3.10* (5.81)	*86.82* *(12.60)*	4.30*** (6.70)	0.27 (1.49)	2.72 (6.47)	2.45 (5.23)	1.88
Neutral	0.16 (1.16)	0.16 (1.16)	1.31* (4.39)	1.20* (3.37)	*96.41* *(7.27)*	0.22 (1.64)	0.05 (0.67)	0.49 (2.19)	0.51
Surprise	3.38** (8.47)	0.05 (0.67)	0.00 (0.00)	0.11 (0.95)	0.16 (1.16)	*93.19* *(10.09)*	1.80* (4.05)	1.31 (3.19)	0.97
Disgust	0.05 (0.67)	0.00 (0.00)	0.05 (0.67)	7.84*** (12.83)	0.11 (0.95)	0.54 (2.07)	*90.52* *(14.05)*	0.87 (2.89)	1.35
With mask									
Fear	*44.61* *(20.80)*	0.38 (1.99)	1.91 (4.11)	1.09 (3.27)	1.25 (4.25)	44.44*** (20.32)	4.63 (7.08)	1.69 (4.81)	7.91
Happiness	0.05 (0.67)	*87.09* *(12.93)*	0.76 (2.59)	1.09 (3.41)	9.04*** (11.39)	0.22 (1.33)	1.09 (2.98)	0.65 (2.79)	1.84
Sadness	8.28*** (10.14)	0.49 (2.19)	*67.05* *(18.45)*	2.72 (5.48)	7.68*** (9.49)	2.51 (5.07)	7.35** (9.93)	3.92 (7.35)	4.71
Anger	0.82 (2.83)	0.33 (1.88)	2.12 (4.33)	*86.49* *(12.20)*	1.96 (4.85)	0.54 (2.47)	6.48*** (8.67)	1.25 (3.42)	1.93
Neutral	0.33 (1.62)	0.33 (1.62)	1.96** (5.04)	1.42 (3.80)	*94.06* *(8.69)*	0.76 (2.59)	0.27 (1.49)	0.87 (3.19)	0.85
Surprise	9.97*** (12.21)	1.20 (3.50)	0.76 (2.59)	0.27 (2.23)	3.21 (7.35)	*80.50* *(16.06)*	2.07 (4.71)	2.02 (4.78)	2.79
Disgust	0.44 (1.86)	6.75 (8.21)	1.74 (4.75)	60.13*** (19.26)	1.47 (3.72)	0.98 (3.44)	*26.91* *(20.20)*	1.58 (4.66)	10.44

The cells show raw choice percentages instead of the analyzed arcsine transformed percentages. Hit rates are marked in italic. Asterisks indicate confusion percentages above the chance level shown in the right column (* *p* < .05, ** *p* < .01, *** *p* < .001). Chance levels refer to the distribution of confusions only, excluding the hits. Percentages are rounded to two digits and may not add up to exactly 100%

The significant mask-by-emotion interaction, *F*(6,906) = 133.67, *p* < 0.001, *η*_p_^2^ = 0.47, indicated that the effect of masks was again larger for some emotions than for others (see Table 3). Excluding actor gender and participant gender, the analyses revealed that the largest reduction in FER again occurred for disgust (minus 64%), *t*(152) = 39.02, *p* < 0.001, *dz* = 3.15. The reduction was also significant for fear (minus 30%), *t*(152) = 16.94, *p* < 0.001, *dz* = 1.37, for sadness (minus 27%), *t*(152) = 22.02, *p* < 0.001, *dz* = 1.78, for surprise (minus 13%), *t*(152) = 11.59, *p* < 0.001, *dz* = 0.94, for happiness (minus 12%), *t*(152) = 14.04, *p* < 0.001, *dz* = 1.14, and for the neutral expression (minus 2%), *t*(152) = 3.63, *p* < 0.001, *dz* = 0.29, but not for anger (minus 0.3%), *t*(152) = 0.32, *p* = 0.751, *dz* = 0.03. The rank order of these reductions was very similar to the rank orders observed before, except that anger and the neutral expression, the two expressions with the smallest mask effects, switched places.

### Confusions

Table 3 also shows the incorrect responses, that is, the confusions. Their distribution was very similar to those of the previous experiments. Again, the confusions were not randomly distributed for any of the masked emotions (see the significance values in the lower half of Table 3). In addition, masked fear was again frequently misinterpreted as surprise (approx. 44%). This confusion occurred as often as the correct interpretation (approx. 44%). Conversely, the most frequent misinterpretation of masked surprise was fear again (approx. 10%). As before, this did not occur nearly as often as the opposite confusion, replicating the asymmetric pattern found before, *t*(152) = 14.28, *p* < 0.001, *dz* = 1.15. Once more, the dominant interpretation of masked disgust was the misinterpretation as anger (approx. 60%), which again occurred much more often than the correct interpretation (approx. 27%), *t*(152) = 10.45, *p* < 0.001, *dz* = 0.84. Conversely, the most frequent misinterpretation of masked anger was disgust (approx. 6%). As before, masked disgust was taken for anger much more often than masked anger was taken for disgust, *t*(152) = 24.57, *p* < 0.001, *dz* = 1.99.

The reciprocal confusion of sadness and the neutral expression (observed in Experiment 1) also occurred: "Sadness" was the only above-chance misinterpretation of the masked neutral expression (approx. 2%), and "neutral" was an above-chance misinterpretation of masked sadness (approx. 8%). However, as in Experiment 2A, "fear" was also a frequent misinterpretation of masked sadness (approx. 8%).

### Experience with face masks

As in Experiment 2A, we did not find a significant correlation of the number of masks participants reported having seen with their FER performance for masked faces or unmasked faces, or with the size of the detrimental effect of masks, all *r*(149) < 0.11, *p* > 0.24.

## Experiments 2A and 2B: Discussion

The goal of Experiments 2A and 2B was to test whether the main findings of the first experiment could be replicated with a new set of facial stimuli taken from the RaFD and with new participant groups. In fact, they were replicated almost perfectly. As in Experiment 1, face masks reduced FER by more than 20%, with the largest detrimental effect observed for disgust, followed by fear, sadness, surprise, and happiness, with only very small effects for the neutral expression and for anger. The confusions observed in Experiments 2A and 2B also mirrored those observed earlier: Again, fear and surprise were confused with each other, as were anger and disgust. The unexpected asymmetry in these confusions was also observed again: Masked fear was misinterpreted as surprise more often than the other way around, and masked disgust was misinterpreted as anger more often than the other way around. The replication of this finding supports the notion that there are indeed reliable response biases that favor the interpretation of ambiguous facial expressions as surprise and anger over fear and disgust, respectively. In contrast, we did not find clear-cut evidence for reciprocal confusions of sadness and the neutral expression. Neither did we find any evidence for a possible dependence of the detrimental mask effect on actor gender, participant gender, or previous experience with face masks.

## General discussion

The three experiments reported here were designed to assess the detrimental effect of face masks on facial emotion recognition (FER), with a particular focus on which emotions would be most affected, and which emotions would be confused with each other. For the latter question, we predicted that participants would confuse emotions that share activation of the muscles that remain visible even when a mask is worn, that is, muscles in the upper part of the face, mainly the forehead and the eyes. To test this hypothesis, it was advantageous that we could use stimuli from the Radboud Faces Database (RafD; Langner et al., 2010), which were created by instructing and training actors to contract emotion-specific patterns of face muscles. This yielded the prediction that anger and disgust would be confused with each other, as would fear and surprise.

The results of the three experiments were remarkably consistent, replicating the main findings. First, the overall detrimental effect of face masks on FER was very similar in all experiments: On average, the percentage of correct identifications was reduced from 88 to 68%. This reduction by 20% is close to the values found earlier, for instance, the 17% reported by Carbon (2020). Although the reduction was highly significant, it should be noted that even with masks, performance was well above chance level (12.5%), also replicating earlier findings (see Pavlova & Sokolov, 2022). Thus, participants were able to use the remaining visible parts of the face during FER. Second, in all experiments the impairment in FER was large for disgust (averaging 60%), followed by fear (31%), sadness (27%), surprise (14%), and happiness (10%), but it was very small for anger (3%) and the neutral expression (2%). The exact values found here may differ from those reported earlier, for instance, we found larger impairments than Carbon (2020) for fear and smaller impairments for happiness. However, one finding stands out as strikingly similar across many studies (see Pavlova & Sokolov, 2022): Disgust is affected most strongly; in our studies so much that misinterpretations as anger were more frequent than correct identifications.

In addition, our exploratory analyses suggest that the mask effect was of similar size for male and female actors, as the mask-by-actor-gender interaction was not significant in any of the experiments. Similarly, Experiment 2B with its larger sample did not yield evidence for a moderating effect of participant gender. Instead, all three experiments yielded a significant emotion-by-mask-by-actor-gender interaction, suggesting that masks impaired FER of all emotions shown by females, but not all emotions shown by males. It is possible that this interaction is due to particular features of the current stimuli taken from the RaFD database, so it should be replicated with stimuli from a different database before speculating about potential explanations. Moreover, it should be kept in mind that the experiments were not specifically designed to test these interactions. For that goal, future experiments should employ more different actor pictures and equal numbers of female and male participants, respectively. Finally, the unexpected negative correlation between mask experience and FER performance observed in Experiment 1 was not replicated in the other experiments, so it may have been a false-positive, or specific to the participant sample of Experiment 1.

As predicted, when participants misinterpreted masked emotions, they frequently confused anger with disgust, and fear with surprise, and this also occurred in all experiments. In addition, they displayed response biases in these confusions: They frequently misinterpreted disgust as anger, fear as surprise, and sadness as neutral, whereas the opposite confusions were less frequent. In Experiment 1, this result was unexpected, but it was replicated in Experiments 2A and 2B. Moreover, these findings are in line with exploratory analyses of confusions in recent research. Carbon (2020) and Noyes et al. (2021) showed higher confusion rates for disgust as anger than for anger as disgust; and Noyes et al. (2021), and Ruba and Pollak (2020) showed that misinterpreting fear as surprise was particularly likely—but not the other way around. In contrast, this pattern converges only partly with confusions of non-masked facial expressions. Both the validation study of the current stimuli set and a study using blurred images showed that disgust is more often mistaken for anger than the other way around, but fear and surprise are both confused with each other (Langner et al., 2010; Wang et al., 2019).

We can only speculate about the potential reasons why surprise was a more likely response than fear, and anger more likely than disgust. It cannot be a general tendency to preferentially process threat-related emotions, otherwise fear should have been a more likely response than surprise (see Hedger et al., 2016). Maybe participants had a tendency to respond with more self-related interpretations: They may find it more likely that actors express anger or surprise because these can be elicited by the perceiver, while disgust and fear may be seen as more likely to be caused by other stimuli. Another possibility may be that participants tended to choose expressions that are more likely to be encountered in everyday life. There is some evidence suggesting that anger and surprise are indeed encountered more frequently than disgust and fear, respectively (Calvo et al., 2014).

In response to the debate on the universality of emotion recognition (e.g., Jack, 2016), it should be noted that the current experiment used Caucasian models and almost all participants resided in Western Europe. In general, emotion recognition seems more accurate for observer and expressor groups with higher exposure to each other (Elfenbein & Ambady, 2002), so exact rates may differ in situations with expressers and observers from more distinct cultural or regional groups. In Europe and the USA, face covering outside medical settings was relatively uncommon before COVID-19, which was reflected in the very limited experience that the participants of Experiment 1 had with masks. As a result, the effect of face masks on emotion recognition in this sample may differ from the effects for people living in areas where face coverings were already part of daily life. Hence, the results pertain to societies that have not had longer experience with covering of the lower face. However, both experienced and new observers of masked faces can only use the visible cues of the upper part of the face, and if spontaneous emotion expression follows universal muscle activation patterns (Girard et al., 2015), these cues would remain equal. An interesting follow-up question would thus be to which extent the confusions of masked faces can be reduced by learning.

The results of the current study should be interpreted in light of the limitations that it necessarily has. First, the external validity may be limited because, like many studies before, we used static pictures of emotional facial expressions, a forced choice answer format, and full-intensity levels of the emotions displayed. This facilitates the comparison to other studies, but in real life, emotional expressions are dynamic, often less intense, and open to many different interpretations. Second, we decided to apply masks (which may have been a little larger or smaller than real ones in some cases) to existing stimuli, instead of creating stimuli of people actually wearing real masks, in order to ensure consistent validity, intensity, and genuineness of expressions in the masked and unmasked condition. Although this creates stimuli that may look slightly artificial, it does not seem to interfere with measuring effects on emotion recognition: Grenville and Dwyer (2022) recently showed that masks superimposed on pictures of faces impair emotion recognition similarly to real masks.

Third, FER confusions in real life may have different nuances, due to stimulus artifacts or because expressers wearing masks exaggerate or otherwise change their facial expressions, or because the act of wearing masks may be associated more with some emotions than with others. Addressing the emotion expresser and the situation, the stimuli were void of all contextual information, such as the situation in which the emotional expression would be shown. For instance, stereotypes about people who wear face masks might have an influence on emotion perception beyond the effect of the covering itself. Moreover, the stimuli lacked the additional information normally conveyed by the expresser's voice and body posture. The latter are important sources of information, and in everyday life, their availability may very well reduce the ambiguity induced by face masks (see also Aviezer et al., 2008; Lecker et al., 2020). Therefore, the current study raises a number of questions that should be addressed by future studies. For instance, which additional cues do perceivers use when emotion recognition is hindered by face masks, and do emotion expressers adapt their (non-)verbal communication if they are aware that their facial expressions are ambiguous? And are these additional efforts by expressers and perceivers sufficient to compensate for the detrimental effects of face masks?

## Conclusions

The current limitations and problems notwithstanding, our study complements earlier research by also showing that face masks hinder facial emotion recognition, and most strongly so the recognition of fear and disgust. Thus, the next time you see a masked face that seems to be looking at you angrily, be aware that the person may actually feel disgust rather than anger. The same applies to a masked face that seems to express surprise: The person may actually feel fear. Thus, it would be useful to look for other cues to disambiguate the expressed emotion. Transparent face masks—if safe enough—could also solve the problem identified here, and they would also help perceivers with hearing problems who depend on lip reading. In any case, the observed detrimental effects of face masks on facial emotion recognition should not be interpreted as sufficient reason not to wear them. While we have to live with the threat of COVID-19, face masks reduce not only the ability to recognize facial expressions of emotion, particularly fear and disgust, but also the spread of SARS-CoV-2.

## Data Availability

Experiments 1 and 2A were pre-registered at the Open Science Framework (1: https://osf.io/jwmkv/), 2A: https://osf.io/86nm7/). The materials, anonymized raw data, and analysis scripts of all three experiments are available there or directly from the authors.
